# Molecular Characterization of the Interplay between *Fasciola hepatica* Juveniles and Laminin as a Mechanism to Adhere to and Break through the Host Intestinal Wall

**DOI:** 10.3390/ijms24098165

**Published:** 2023-05-03

**Authors:** Judit Serrat, María Torres-Valle, Marta López-García, David Becerro-Recio, Mar Siles-Lucas, Javier González-Miguel

**Affiliations:** Laboratory of Helminth Parasites of Zoonotic Importance (ATENEA), Institute of Natural Resources and Agrobiology of Salamanca (IRNASA-CSIC), C/Cordel de Merinas 40-52, 37008 Salamanca, Spain; judit.serrat@irnasa.csic.es (J.S.); maria.torres@irnasa.csic.es (M.T.-V.); marta.lopez@irnasa.csic.es (M.L.-G.); david.becerro@irnasa.csic.es (D.B.-R.); mmar.siles@irnasa.csic.es (M.S.-L.)

**Keywords:** *Fasciola hepatica* newly excysted juveniles, fasciolosis, host–parasite relationships, extracellular matrix, laminin, adhesion, migration

## Abstract

*Fasciola hepatica* is the main causative agent of fasciolosis, a zoonotic parasitic disease of growing public health concern. *F. hepatica* metacercariae are ingested by the host and excyst in the intestine, thereby releasing the newly excysted juveniles (FhNEJ), which traverse the gut wall and migrate towards the biliary ducts. Since blocking *F. hepatica* development is challenging after crossing of the intestinal wall, targeting this first step of migration might result in increased therapeutic success. The intestinal extracellular matrix (ECM) is constituted by a network of structural proteins, including laminin (LM) and fibronectin (FN), that provide mechanical support while acting as physical barrier against intestinal pathogens. Here, we employed ELISA and immunofluorescent assays to test for the presence of LM- and FN-binding proteins on a tegument-enriched antigenic fraction of FhNEJ, and further determined their identity by two-dimensional electrophoresis coupled to mass spectrometry. Additionally, we performed enzymatic assays that revealed for the first time the capability of the juvenile-specific cathepsin L3 to degrade LM, and that LM degradation by FhNEJ proteins is further potentiated in the presence of host plasminogen. Finally, a proteomic analysis showed that the interaction with LM triggers protein changes in FhNEJ that may be relevant for parasite growth and adaptation inside the mammalian host. Altogether, our study provides valuable insights into the molecular interplay between FhNEJ and the intestinal ECM, which may lead to the identification of targetable candidates for the development of more effective control strategies against fasciolosis.

## 1. Introduction

*Fasciola hepatica* is the most common causative agent of fasciolosis, and it has a complex life cycle that includes an intermediate snail host, where the asexual phases of the parasite multiply, and a definitive mammalian host, where the parasites develop into sexually mature flukes. Definitive hosts, usually ruminants and humans, become infected upon ingestion of aquatic plants or water contaminated with metacercariae, which release the newly excysted juveniles (FhNEJ) upon arrival to the duodenum. Shortly after excystment, FhNEJ cross the intestinal wall and migrate through the peritoneum to eventually penetrate the liver. At this point, immature flukes initiate a migration period through the liver parenchyma until they finally gain access into the major hepatic biliary ducts, where adult parasites develop, and egg shedding starts [1]. By infecting millions of ruminants worldwide, *F. hepatica* infection causes major economic losses in the livestock industry [2] and represents a major threat to animal welfare and food security [3]. In addition, current estimations on the prevalence of human fasciolosis reveal that up to 17 million people may be infected worldwide [4]. Since human infections are particularly concentrated in regions where people live in substandard economic conditions, human fasciolosis is considered a neglected foodborne zoonotic disease of growing public health concern [5]. 

Increasing reports of human and veterinary cases of fasciolosis that are resistant to the standard drug, triclabendazole, together with the lack of an effective vaccine, raise awareness on the need to find alternative treatment and control strategies against *F. hepatica* [1]. Given that crossing of the intestinal wall by FhNEJ is considered a ‘point of no return’ in fasciolosis in terms of therapeutic control [6], a detailed understanding on the processes that drive FhNEJ trans-intestinal migration may be useful in this regard. 

The intestinal mucosa represents the first and most extensive line of defence against pathogens that gain access to the mammalian organism through the intestinal tract. Structurally, this tissue is formed by a monolayer of diversely specialized epithelial cells that sit on top of the intestinal extracellular matrix (ECM), which is externally outlined by a thin layer of smooth muscle cells [7]. The intestinal ECM consists of an acellular mesh of structural proteins that give mechanical support to the tissue while acting as a physical barrier against invading pathogens [7,8,9]. In addition to physically blocking pathogen entry, the intestinal mucosa is endowed with potent immune mechanisms to prevent pathogen invasion [10], and beyond its supportive and defensive roles, the ECM is also regarded as a master regulator of cell activity by triggering intracellular signalling events that regulate cell differentiation and migration [11].

The intestinal ECM is divided in two sub-compartments. Sitting right beneath the epithelial layer we find the basement membrane, which is mainly formed by collagen type IV, laminin (LM), nidogens, and perlecan; immediately below the basement membrane, there is the interstitial layer, whose major components are collagen I and III, fibronectin (FN) and elastin [7]. In order to break through the intestinal epithelium and succeed at host invasion, intestinal pathogens have evolved a myriad of mechanisms to overcome the intestinal ECM and evade immune responses that are initiated in this tissue towards them [8,9]. A paradigmatic example of such mechanisms is the expression of ECM-binding proteins (collectively termed adhesins) and proteases by intestinal pathogenic bacteria that bind to and degrade a variety of ECM components [8,12]. The importance of ECM-binding and degrading proteins for successful tissue invasion is evidenced by studies showing that blocking these factors results in a dramatic reduction in the invasive capacity of pathogens [8]. In addition to endogenous proteases, many intestinal pathogens hijack the functions of the host fibrinolytic enzyme plasmin, which is a broad-spectrum serine protease that degrades several components of the intestinal ECM [13], for colonization purposes. The ability of degrading ECM components both by endogenous proteases and by an induction of plasmin generation from the host-circulating zymogen plasminogen (PLG) is a widespread phenomenon in bacterial, fungal, and protozoan pathogens [8], but remains largely unexplored in helminth parasites, including *F. hepatica*.

Here, we employed a combination of enzyme linked immunosorbent (ELISA), immunofluorescence and enzymatic assays to study the interaction between FhNEJ and LM, one of the major components of the intestinal basement membrane, and we further analysed the consequences of the FhNEJ-LM crosstalk at a proteomic level to better understand the biological relevance of this event in the establishment of infection. Collectively, these results broaden our knowledge on host–parasite relationships in early fasciolosis, which may pave the way for the identification of attractive targets for future anti-*Fasciola* therapeutics.

## 2. Results

### 2.1. The FhNEJ Tegument-Enriched Protein Fraction (FhNEJ-Teg) Contains Proteins That Bind to LM but Not FN

**Figure 1:**
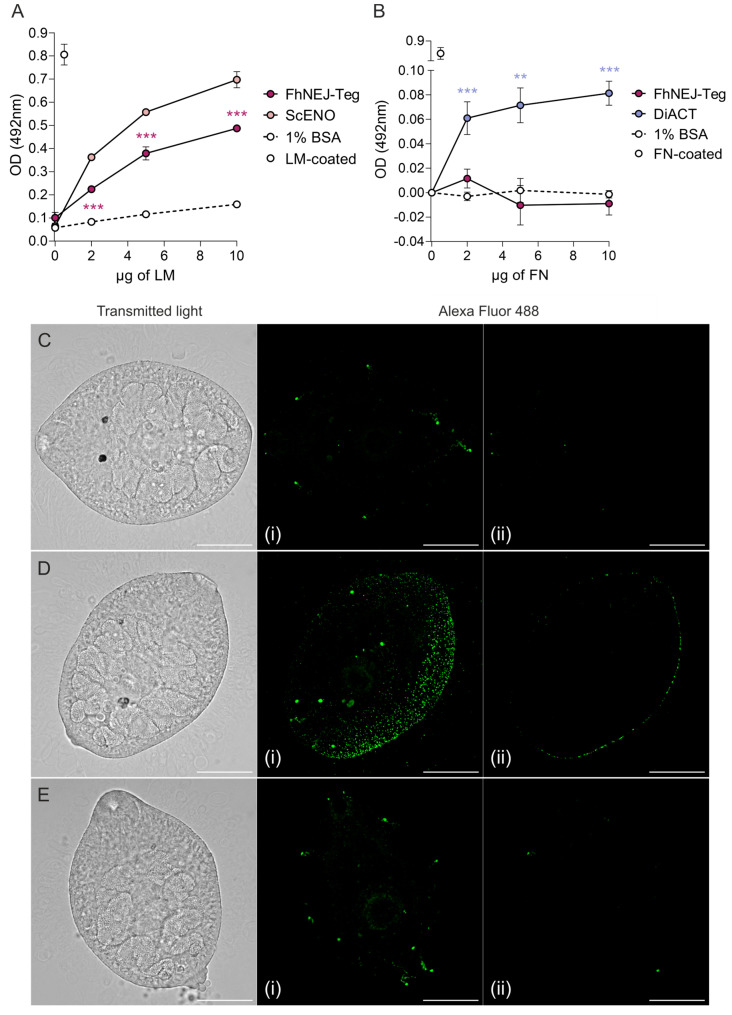
FhNEJ-Teg contains proteins that interact with LM, but not FN. Binding of FhNEJ-Teg proteins to either LM (**A**) or FN (**B**) was detected via ELISA by coating wells with FhNEJ-Teg followed by incubation with increasing amounts of LM or FN. ScENO and DiACT served as positive controls for LM and FN binding, respectively, and 1% BSA was used as a negative control for unspecific binding of both ECM proteins. Optical densities (OD) were measured at 492 nm. Dots indicate the mean of three technical replicates ± SD. Asterisks indicate significant differences between the corresponding group and the negative control (** *p* ≤ 0.01; *** *p* ≤ 0.001; one-way ANOVA). (**C**–**E**) FhNEJ were whole-mounted and LM binding on their surface (Alexa Fluor 488) was detected on a confocal laser microscope. Panels show representative images of FhNEJ incubated in the absence (**C**) or presence (**D**) of 100 µg/mL of LM or with secondary antibody alone (**E**). A maximum projection of all acquired planes spanning the entire FhNEJ surface (**i**), and the middle plane of each projection (**ii**) are shown to highlight specific staining at the parasite surface. Scale bars, 50 µm.

First, after in vitro excystment of *F. hepatica* metacercariae, FhNEJ isolation, and extraction of their tegument-enriched protein fractions, we analysed whether FhNEJ-Teg contains proteins that are capable of interacting with two proteins of the intestinal ECM, such as LM and FN. To this end, we set up an ELISA-based experiment in 96-well microtiter plates by coating wells with 1 µg of FhNEJ-Teg or 1% bovine serum albumin (BSA) as a control for unspecific binding. Upon incubation of wells with increasing amounts of LM, we observed that binding of LM by FhNEJ-Teg proteins is concentration-dependent, statistically greater than that obtained in wells coated with 1% BSA and similar to that mediated by our positive control, a recombinant *Saccharomyces cerevisae* enolase (ScENO) (Figure 1A). In parallel to this, a similar experiment was performed by adding human FN instead of LM to FhNEJ-Teg-coated wells to analyse FN binding by FhNEJ-Teg proteins. Interestingly, this experiment showed that FhNEJ-Teg does not contain proteins that bind to FN (Figure 1B). In parallel to this, LM binding on the surface of FhNEJ was also confirmed by immunofluorescent staining of whole-mounted parasites (Figure 1C,D), which revealed specific binding of this ECM protein all over the FhNEJ surface.

### 2.2. Identification of Potential LM-Binding Proteins in FhNEJ-Teg

**Figure 2:**
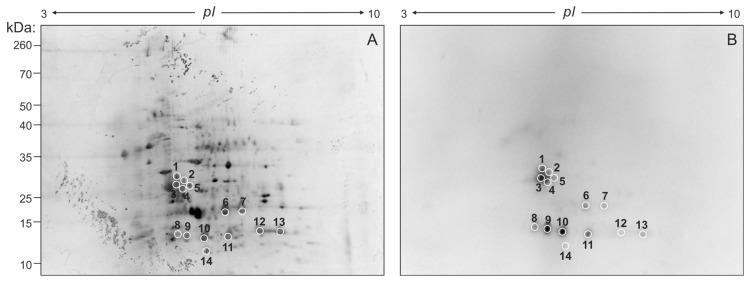
2D electrophoresis of FhNEJ-Teg and detection of LM-binding proteins by immunoblotting. 40 µg of FhNEJ-Teg were separated in two dimensions using IPG strips with a pH range of 3–10 and 12% SDS-PAGE gels. This procedure was performed in duplicate so that the proteins in one of the gels were revealed by silver staining (**A**) and the others were transferred to a nitrocellulose membrane for detection of LM binding by immunoblotting (**B**). LM-binding protein spots are circled and numbered.

**Figure 3:**
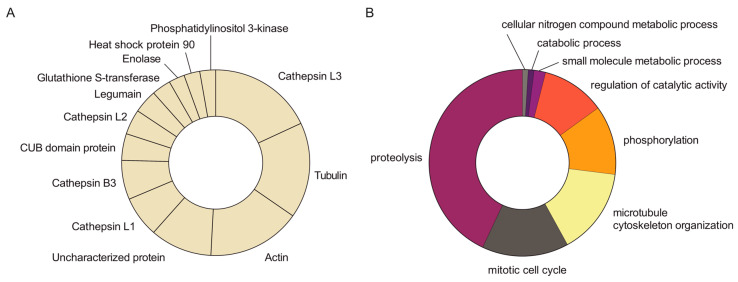
MS analysis of potential LM-binding proteins contained within LM-reactive spots. Protein spots detected in the silver-stained gel containing LM-binding proteins were manually excised and analysed by MS. A total of 14 protein spots were identified, containing 80 different proteins with an average of 16 proteins per spot. (**A**) Abundance of the most recurrently identified proteins in the pool of potential LM-binding proteins. (**B**) GO analysis under the Biological Process category of the potential LM-binding proteins identified by MS.

Aiming at identifying the proteins contained within FhNEJ-Teg that are involved in LM binding, we first separated the proteins contained within this extract by two-dimensional (2D) electrophoresis. Separation was performed in two gels so that the protein spots in one of the gels were revealed by silver staining (Figure 2A) and the proteins contained in the other gel were transferred to a nitrocellulose membrane to detect LM binding by standard Western Blot procedures (Figure 2B). After matching the LM-reactive spots that appeared in the blot with those in the homologous silver-stained gel, we detected a total of 14 protein spots that were sent for analysis by mass spectrometry (MS). Based on similarity to *F. hepatica* sequences contained in the Uniprot_trematoda database, we identified a total of 80 proteins in FhNEJ-Teg with potential to bind LM and an average of 16 proteins per spot (Table S1). Figure 3A shows the proteins that were most recurrently identified and Figure 3B represents the Gene Ontology (GO) annotation under the Biological Process category of all the potential LM-binding proteins identified by 2D-MS. *F. hepatica* cathepsin L3 (FhCL3) stands out as the protein that is most recurrently identified within LM-reactive spots, and GO annotation reveals that the identified potential LM-binding proteins contained within FhNEJ-Teg are involved in proteolysis, mitotic cell cycle, microtubule cytoskeleton organization, phosphorylation, regulation of catalytic activity, small molecule metabolic process, catabolic process, and cellular nitrogen compound metabolic process. 

**Figure 4:**
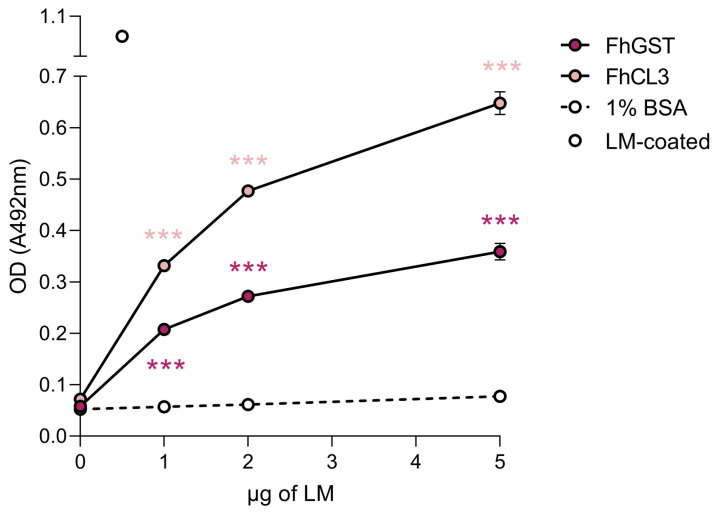
FhCL3 and FhGST bind to LM in a concentration-dependent manner. The capability of FhCL3 and FhGST to bind to LM was assessed via ELISA using recombinant versions of these proteins. Wells coated with 1% BSA served as negative controls for unspecific LM binding. Optical densities (OD) were measured at 492 nm. Dots indicate the mean of three technical replicates ± SD, and asterisks indicate significant differences between the indicated protein and the negative control (*** *p* ≤ 0.001; one-way ANOVA).

We next sought to validate our 2D-MS results via ELISA-based LM-binding assays using recombinant versions of FhCL3 and *F. hepatica* glutathione S-transferase (FhGST). To this end, we coated microtiter plate wells with the abovementioned recombinant proteins in a neutral buffer that preserves the proteins’ native conformations and incubated them with increasing amounts of LM, which confirmed that FhCL3 and FhGST are capable of binding LM in a concentration-dependent manner and to a significantly greater extent than that showed by the negative control, 1% BSA (Figure 4).

### 2.3. LM Degradation by FhCL3

**Figure 5:**
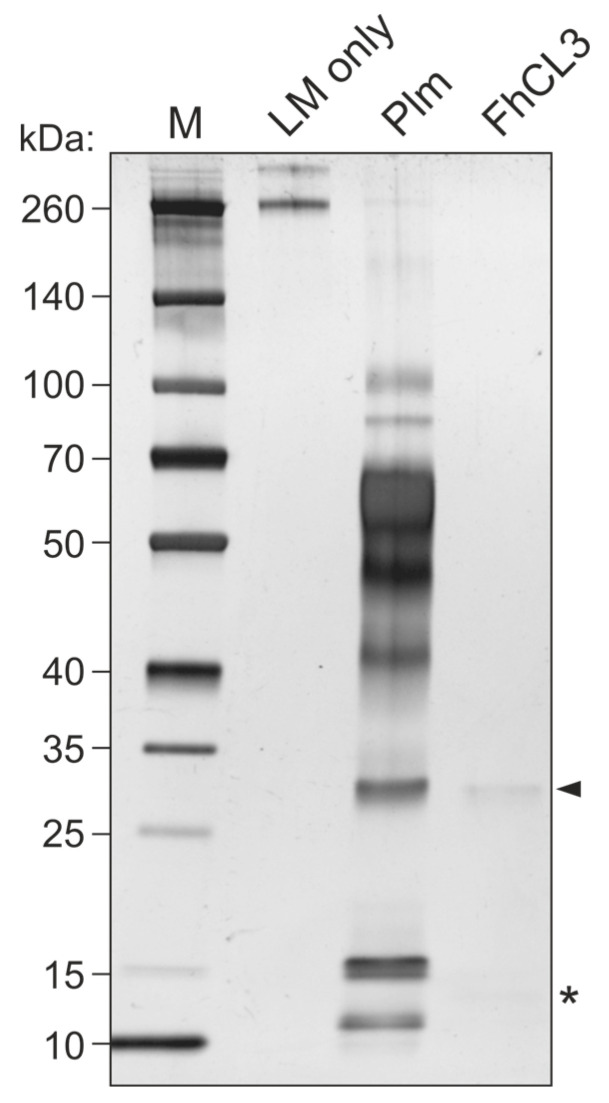
LM degradation by FhCL3. Mature FhCL3 (1 µM) was incubated with LM (0.1 μg) for three hours at 37 °C and LM degradation was assessed by SDS-PAGE and silver staining. A condition containing an equivalent amount of plasmin (Plm) in place of FhCL3 was used as a positive control for LM degradation. Lane 1, MW marker; lane 2, LM only; lane 3, LM + Plm; lane 4, LM + mature FhCL3; 4–20% polyacrylamide gel stained with silver. Arrowhead indicates mature FhCL3, asterisk indicates FhCL3 pro-peptide.

Based on our observation that FhCL3 is capable of binding LM, we next tested whether this protein would also be proteolytically active towards this ECM component. To this end, we set up an enzymatic assay where we co-incubated mature FhCL3 with LM for three hours to assess LM degradation by this enzyme. Auto-catalytic activation of FhCL3 was performed and confirmed by running the activated enzyme mix on a 12% SDS gel prior to incubation with LM, which revealed a ~35 kDa band corresponding to the mature enzyme and a ~12 kDa band corresponding to the protease pro-peptide (Figure S1). Incubation of mature FhCL3 with LM resulted in complete fading of protein bands corresponding to LM comparable to that shown by our positive control, plasmin (Figure 5). 

### 2.4. FhNEJ-Teg Contains Proteins That Act as Cofactors for Plasmin-Mediated Degradation of LM

**Figure 6:**
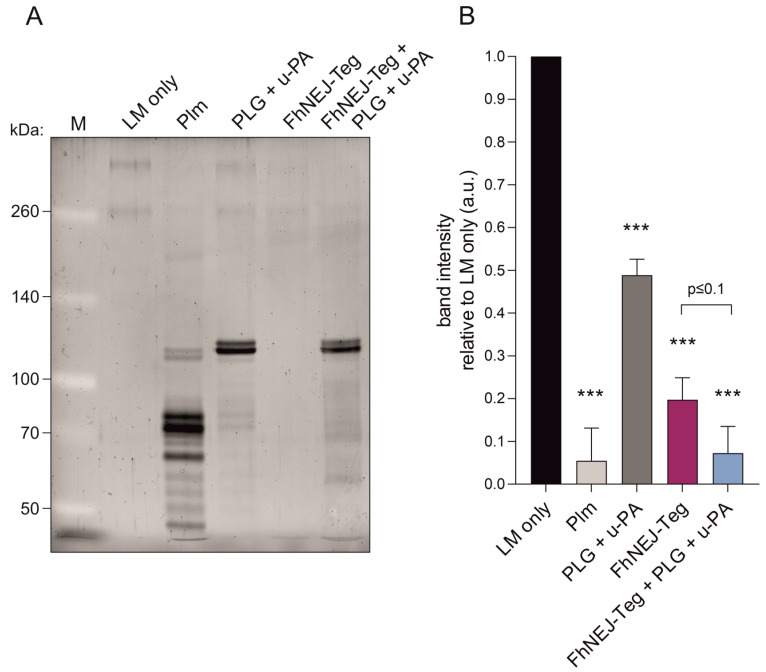
FhNEJ-Teg contains proteins that act as cofactors for plasmin-mediated LM degradation. LM was incubated either alone or with FhNEJ-Teg in the presence or absence of PLG and u-PA and incubated for three hours at 37 °C. LM incubated with Plm served as a positive control for LM degradation; and LM incubated with PLG + u-PA (without FhNEJ-Teg) was used to control for background LM degradation derived from u-PA-induced Plm generation from soluble PLG. (**A**) Representative image of three independent experiments. Lane 1, MW marker; lane 2, LM only; lane 3, LM + Plm; lane 4, LM + PLG + u-PA, lane 5, LM + FhNEJ-Teg; lane 6, LM + FhNEJ-Teg + PLG + u-PA; 6% polyacrylamide gel stained with Sypro Ruby. (**B**) Quantification of LM degradation. Bars indicate the mean of three independent experiments ± SD, and asterisks indicate significant differences between each condition and lanes containing LM only (*** *p* ≤ 0.001; one-way ANOVA).

We have recently shown that FhNEJ-Teg contains proteins that interact with the host fibrinolytic system by binding PLG and promoting its conversion into the active protease, plasmin [14], which is a broad-spectrum serine protease that degrades multiple substrates, including LM [15,16]. Based on this, we sought to determine whether FhNEJ-Teg-induced plasmin generation from bound PLG could potentiate LM degradation by mixing FhNEJ-Teg with PLG, the urokinase-type PLG activator (u-PA), and LM. In some instances, FhNEJ-Teg, PLG, and u-PA were omitted, and plasmin was added as a positive control for LM degradation. After incubation for three hours at 37 °C, the samples were loaded into 6% polyacrylamide gels for visualization of LM degradation. This experiment showed that LM degradation is enhanced in the presence of FhNEJ-Teg proteins together with PLG and u-PA, confirming that PLG-binding proteins contained within the FhNEJ-Teg extract promote LM degradation by stimulating plasmin generation from bound PLG. Remarkably, FhNEJ-Teg in the absence of PLG and u-PA also gave a significant decrease in LM band intensity as compared to control lanes containing LM, PLG, and u-PA (Figure 6).

### 2.5. Proteomic Changes Induced in FhNEJ upon Interaction with LM

In order to study whether the interaction with LM triggers proteomic changes in FhNEJ, we incubated live FhNEJ in plates coated with or without LM and isolated both FhNEJ-Teg and an FhNEJ somatic-enriched antigenic fraction (FhNEJ-Som) to perform a quantitative analysis of differentially expressed proteins (DEPs) by Sequential Window Acquisition of All Theoretical Mass Spectra (SWATH-MS). This analysis identified a total of 495 and 302 proteins in FhNEJ-Teg and FhNEJ-Som, respectively. Discriminant Analysis showed that samples of each experimental condition clustered together (Figure S2), and the analysis revealed that incubation with LM resulted in overexpression of 5 and 14 proteins in FhNEJ-Teg and FhNEJ-Som, respectively, and downregulation of one protein in both antigenic compartments (Table 1). Among others, we identified a member of the GST family of proteins to be overexpressed in FhNEJ-Teg upon incubation with LM; and we also detected an overexpression of the juvenile-specific cathepsin B2 (FhCB2) in FhNEJ-Som of LM-incubated FhNEJ compared to their control counterparts. Overexpression of FhCB2 in FhNEJ-Som upon culture in LM-coated plates was validated by Western Blot (Figure S3).

## 3. Discussion

In order to characterize the molecular mechanisms used by FhNEJ to cross the intestinal wall and initiate the migration process that will eventually drive them to the liver, we first sought to analyse whether this parasitic stage is capable of specifically interacting with components of the intestinal ECM, namely LM and FN. Given that the tegument, the outermost layer of the parasite, is constantly exposed to host tissues, thereby representing the host–parasite interface [6], we tested for the presence of LM- and FN-binding proteins in a tegument-enriched protein fraction of FhNEJ (FhNEJ-Teg). This experiment showed that FhNEJ-Teg is capable of binding LM, but not FN, in a concentration-dependent manner. Since LM is a major component of the intestinal basement membrane, which is the tissue layer that sits immediately underneath the intestinal epithelium, whereas FN is found in deeper tissue layers, such as the interstitial matrix [7], these results suggest that FhNEJ are specifically adapted to interact with the tissue microenvironment that they encounter immediately after excystment. 

We next sought to determine the identity of the proteins contained within FhNEJ-Teg that are involved in LM binding by 2D electrophoresis and ligand blotting coupled to MS, which revealed the presence of 80 potential LM-binding proteins in this antigenic extract. The juvenile-specific FhCL3 stands out as the protein that is most recurrently identified in the pool of LM binding proteins, together with tubulin, actin, cathepsin L1, FhCB3, CUB domain protein, cathepsin L2, legumain, FhGST, enolase, heat-shock protein 90, and phosphatidylinositol 3-kinase. Since 2D-MS is performed under denaturing conditions, which could potentially expose internal LM-binding motifs in proteins that are not used for LM binding in a real physiological context, we validated some of these results by ELISA, which is performed under native conditions. We focused on FhCL3 and FhGST given their biological importance during the early immature stage of the parasite [17,18,19] and confirmed that these proteins are indeed capable of binding to LM specifically and in a concentration-dependent manner. To the best of our knowledge, this is the first time that an ECM binding function is described for FhCL3 and FhGST, supporting a role for these proteins as adhesins [12]. 

Next, we performed a functional annotation analysis on the potential LM-binding proteins that we identified via 2D-MS and observed that these proteins are mainly involved in biological processes of proteolysis, mitotic cell cycle, microtubule cytoskeleton organization, phosphorylation, regulation of catalytic activity, small molecule metabolic process, catabolic process, and cellular nitrogen compound metabolic process. The fact that some of the FhNEJ-Teg proteins that interact with LM are related to cytoskeleton organization is compelling considering that ECM components, including LM, are important triggers of intracellular signalling events that culminate in remodelling of the cellular cytoskeleton, which is required for migration of cells across tissues [20,21,22,23]. Furthermore, cell proliferation at the juvenile stage of the parasite is particularly important to sustain parasite growth and development, which is mainly supported by stem cell-like cells known as neoblasts [24] and the phosphatidylinositol 3 kinase (PI3K)-protein kinase B (Akt) signalling pathway [25]. Interestingly, LM has been shown to stimulate the PI3K-Akt pathway, which in turn enhances fibroblast proliferation [26]. Altogether, these results suggest that the interaction between FhNEJ and LM may not simply serve as an anchoring/adhesion mechanism to the intestinal mucosa, but also operate as a spatial cue that triggers intracellular molecular events in FhNEJ related to parasite migration and development inside the mammalian organism, which most certainly deserves further attention. 

FhCL3 is regarded as a master driver of FhNEJ migration through the intestinal wall owing to its capability to degrade collagen [27,28,29], a major component of this tissue layer together with LM [7]. In view of this and considering that this protein was most recurrently identified as an LM-binding protein in our 2D-MS analysis, we next tested whether FhCL3 would also have the ability to degrade bound LM, as described for cathepsin L isoforms expressed by adult flukes [30,31]. Our results revealed that mature FhCL3 is indeed capable of hydrolysing LM to a similar or event greater extent as our positive control, the main fibrinolytic protease plasmin. Since LM integrity is maintained under acidic conditions [32], we are confident that the observed LM degradation is not due to loss of LM structure under the specific assay reactions that we used, and that LM degradation by FhCL3 also occurs in a physiological context provided that this protease is most active at less acidic pH values, such as those found in the host duodenum [33]. 

We have recently demonstrated that FhNEJ interact with the host fibrinolytic system by expressing proteins that bind to PLG, which facilitates the conversion of this zymogen into its catalytically active form, plasmin [14]. PLG activation occurs upon binding of PLG to receptors or partner proteins so that a conformational change is induced in the PLG molecule that exposes cleaving sites for the tissue-type and urokinase-type PLG activators (t-PA and u-PA, respectively), which selectively cleave PLG and convert it into its catalytically active form, plasmin [13,34]. Provided that plasmin is a broad-spectrum serine protease that degrades multiple ECM components, including LM [15,35], the stimulation of plasmin generation from bound PLG by FhNEJ-Teg proteins could potentially be used to degrade intestinal ECM components and support FhNEJ migration through the intestinal wall [36,37,38]. Based on this, we set out to analyse whether PLG binding by FhNEJ-Teg would potentiate the endogenous, FhCL3-mediated, capacity of FhNEJ to degrade LM. To this end, we co-incubated FhNEJ-Teg with LM in the presence or absence of PLG and u-PA to analyse whether subsequent LM degradation would be further enhanced in the presence of these fibrinolytic factors. We included u-PA over t-PA in the reaction mixes provided that u-PA is the PLG activator that is selectively expressed by the intestinal epithelium [39]. Although PLG is preferentially activated when bound to its physiologic receptors [34], u-PA is also capable of cleaving soluble (unbound) PLG [40], which resulted in certain background LM degradation due to plasmin generation from unbound PLG (Figure 6, compare lanes 4 and 2). Despite this technical constraint, we confirmed that FhNEJ-Teg contains proteases that degrade LM (Figure 6, compare lanes 5 and 4) and, most interestingly, that this proteolytic activity is further enhanced when the reaction is performed in the presence of PLG and u-PA (Figure 6, compare lanes 6 and 5), which validates our hypothesis that FhNEJ-Teg proteins act as cofactors for plasmin-induced LM degradation through PLG binding and the stimulation of plasmin generation from bound PLG. Collectively, our LM degradation assays reveal that FhNEJ are endowed with a potent and redundant proteolytic machinery that is purposely crafted for degrading the major structural components of the intestinal basement membrane, namely collagen [27,28,29] and LM, which act as a physical barrier that needs to be overcome by FhNEJ for the successful establishment of the parasite within its mammalian host.

Finally, and provided that ECM components are potent triggers of intracellular signalling events in eukaryotic cells [11] and parasites [41,42], we envisioned that binding to LM would not simply be used for adhesion purposes, but that the interaction with this ECM component may also trigger changes in the protein expression profile of FhNEJ. To test this, FhNEJ were added to LM-coated plates for three hours since this is the average time that the parasites take to cross the intestinal wall [4], meaning that proteomic changes induced by LM interaction should occur within this time frame if they were to be relevant in a real physiological setting. After incubation, FhNEJ were harvested and differences in protein expression between LM-incubated and control FhNEJ were quantified by MS. 

Among others, we found differential expression of several proteins related to glucose metabolism upon incubation of FhNEJ with LM. More specifically, we detected an overexpression of glucose 6-phosphate isomerase, the enzyme that catalyses the second step of glycolysis, and downregulation of oxoglutarate dehydrogenase, which is involved in the tricarboxylic acid (TCA) cycle, a metabolic pathway that fuels oxidative phosphorylation. This finding suggests that host LM could function as a molecular trigger of the gradual metabolic switch that is required during parasite growth and development, which limits oxygen diffusion towards deeper tissues and generates a strong dependence on anaerobic metabolism for energy production [6,43]. This idea is apparently conflicting with the fact that we also detected overexpression of enzymes involved in aerobic energy metabolism (e.g., NADH dehydrogenase and alpha-aminoadipic semialdehyde synthase) in FhNEJ upon incubation with LM. However, a downregulation of oxoglutarate dehydrogenase expression would most likely shift FhNEJ metabolism to gradually depend on anaerobic pathways provided that, in humans, a congenital deficiency of this enzyme leads to severe impairment of the TCA cycle and oxidative respiration [44,45]. Of note, increased expression of glycolytic enzymes and downregulation of enzymes of the TCA cycle has also been detected in FhNEJ after crossing the intestinal wall in an ex vivo model of trans-intestinal migration [19]. 

In addition to metabolic changes, we detected an overexpression of FhCB2 in FhNEJ-Som upon incubation with LM. Western blot analysis of FhNEJ-Som confirmed that FhCB2 is overexpressed in FhNEJ upon interaction with LM, which was also interpreted as a validation of our statistical and bioinformatic analyses to identify DEPs. Based on what is known for *Schistosoma mansoni* orthologs [46], FhCBs have been suggested to be involved in the immunomodulatory mechanisms that operate in FhNEJ to control innate immune pathways that would otherwise stimulate adaptive pro-inflammatory responses towards them [47]. Therefore, overexpression of FhCBs upon direct contact with LM, as shown by our results, appears to be particularly important during intestinal crossing given the highly proficient immune mechanisms that operate in this organ [10]. This said, it remains a possibility that additional juvenile-specific cathepsin B clades, namely FhCB1 and FhCB3, are upregulated upon interaction with LM provided their high degree of homology, which hinders the precise annotation of these proteins.

Finally, we also detected overexpression of detoxifying enzymes, such as thioredoxin and FhGST, in FhNEJ upon incubation with LM, which adds an extra layer of biological relevance to FhGST expression during FhNEJ development besides its protective antioxidant functions [48]. The fact that FhGST is specifically overexpressed in FhNEJ-Teg upon incubation with LM, and that our 2D-MS and ELISA-based experiments revealed that FhGST is an LM-binding protein located in this antigenic compartment, advocates for the existence of a positive feedback loop between FhNEJ and the host intestinal ECM aimed at improving FhNEJ adhesiveness to the intestinal mucosa, which might be necessary provided the mechanical force exerted by intestinal fluids that FhNEJ need to resist so as not to be swept down the gastrointestinal tract before gut passage. 

## 4. Materials and Methods

### 4.1. In Vitro Excystment of F. hepatica Metacercariae

An amount of 5000 *F. hepatica* metacercariae (Ridgeway Research Ltd., St Briavels, UK) were excysted in vitro as previously described [49]. In brief, CO_2_ was bubbled in 10 mL of ice-cold distilled water for 30 s, the solution was supplemented with 0.02 M of sodium dithionite, and added to metacercariae for 1 h at 37 °C. Next, metacercariae were washed twice with distilled water, resuspended in 5 mL of Hank’s balanced salt solution (Sigma, St. Louis, MO, USA) supplemented with 10% lamb bile (obtained from a local abattoir) and 30 mM HEPES (Sigma, St. Louis, MO, USA) pH 7.4, and incubated at 37 °C. FhNEJ were manually recovered under a stereomicroscope using a 20 µL pipette and immediately subjected to protein extraction.

### 4.2. Protein Extraction of the Tegument- and Somatic-Enriched Antigenic Fractions of FhNEJ

Upon excystment, FhNEJ were processed as previously described [17,50]. Briefly, FhNEJ were washed twice in sterile phosphate-buffered saline (PBS), resuspended in 200–300 µL of PBS containing 1% Nonidet P-40 (NP-40; Sigma, St. Louis, MO, USA), and incubated at room temperature for 30 min with mild rotation. Following incubation, FhNEJ were centrifuged five min at 300× *g* and the supernatant containing the FhNEJ-Teg was frozen at −80 °C until use. Protein pellets containing naked FhNEJ were resuspended in 200 μL of RIPA buffer (Sigma, St. Louis, MO, USA) and disrupted by ultrasound (five cycles of 30 s). The samples were then centrifuged at 1000× *g* for 5 min and the clarified supernatant containing the FhNEJ-Som was transferred to clean tubes and frozen at −80 °C. Protein concentration was determined using the Pierce BCA Protein Assay kit (Thermo Fisher Scientific, Waltham, MA, USA).

### 4.3. LM and FN Binding Assays

LM and FN binding were assessed by ELISA as previously described [51] with minor modifications. Briefly, microtiter 96-well plates were coated overnight at 4 °C with 1 μg of FhNEJ-Teg or 0.5 µg of recombinant FhCL3 [33] or FhGST (kind gift from Prof. J.P. Dalton) diluted in 200 μL of 15 mM Na_2_CO_3_, 35 mM NaHCO_3_, pH 9.6. 1% BSA-coated wells served as negative controls for unspecific binding of LM and FN. We used recombinant *Saccharomyces cerevisae* enolase (ScENO; Sigma, St. Louis, MO, USA) as a positive control for LM binding as it shares over 50% sequence identity with enolase from *Paracoccidioides brasiliensis*, which has been previously described to bind LM [52], and recombinant actin from the roundworm *Dirofilaria immitis* (DiACT) [53] was used as a positive control for FN binding (unpublished data from our lab). Wells coated with 0.5 µg of FN (Santa Cruz Biotechnology, Dallas, TX, USA) or LM (Santa Cruz Biotechnology, Dallas, TX, USA) of human and mouse origin, respectively, were used as primary antibody controls. Following two washes in PBS containing 0.05% Tween_20_ (PBST), wells were blocked with 1% BSA for one hour at 37 °C and increasing amounts of LM (0 μg to 10 μg per well) or FN (0 μg to 10 μg per well) diluted in blocking solution were added and incubated for two hours at 37 °C. Wells were washed three times in PBST and incubated for two hours at 37 °C with the corresponding primary antibodies diluted in blocking solution, anti-LM raised in rabbit (1:2000; Abcam, Cambridge, UK) and anti-FN raised in mouse (1:500; Santa Cruz Biotechnology, Dallas, TX, USA). Following three washes in PBST, wells were incubated with horseradish peroxidase (HRP)-conjugated anti-rabbit or anti-mouse IgG (Sigma, St. Louis, MO, USA) diluted 1:3000 and 1:2000, respectively, in blocking solution and further incubated for one hour at 37 °C. Finally, wells were washed three times in PBST and LM or FN binding was revealed by adding 100 μL of 1.5 mM of the chromogenic substrate ortho-phenylene-diamine (Sigma, St. Louis, MO, USA) diluted in 25 mM citric acid, 45 mM Na_2_HPO_4_, 0.04% H_2_O_2_, pH 5. The reaction was stopped by adding 100 μL of sulphuric acid 2 N and optical densities (OD) were measured at 492 nm in a Multiskan GO spectrophotometer (Thermo Fisher Scientific, Waltham, MA, USA). LM and FN binding assays were performed in triplicate.

### 4.4. Detection of LM Binding on the Surface of FhNEJ by Immunofluorescence

*F. hepatica* metacercariae were excysted as described in Section 4.1 and FhNEJ were recovered every one hour after addition of excystment medium and transferred to plates containing RPMI-1640 culture media (Thermo Fisher Scientific, Waltham, MA, USA) supplemented with 30 mM HEPES (Sigma, St. Louis, MO, USA), 0.1% glucose (Sigma, St. Louis, MO, USA), and 50 μg/mL gentamycin (Sigma, St. Louis, MO, USA) at 37 °C in a 5% CO_2_ atmosphere [54]. One hour after recovery, 80 FhNEJ per condition were washed three times in PBS and incubated with blocking solution (0.1% BSA in PBS) supplemented with 100 µg/mL of LM (Santa Cruz Biotechnology, Dallas, TX, USA) for two hours at 37 °C. Two extra sets of FhNEJ incubated in blocking buffer alone served as negative controls for LM binding and to control for unspecific background staining derived from the secondary antibody. After LM incubation, FhNEJ were fixed in 4% paraformaldehyde (Santa Cruz Biotechnology, Dallas, TX, USA) for one hour at room temperature and probed with anti-LM antibody raised in rabbit (Abcam, Cambridge, UK) diluted 1:100 followed by incubation with Alexa Fluor 488 donkey anti-rabbit IgG (Thermo Fisher Scientific, Waltham, MA, USA) diluted 1:500. FhNEJ were washed three times with blocking buffer between incubations and primary and secondary antibodies were diluted in blocking solution and incubated overnight at 4 °C. Finally, FhNEJ were washed once in blocking solution, twice in PBS and whole-mounted in glass slides using a 9:1 glycerol solution containing 0.1 M propyl gallate. Specimens were viewed using a spinning disk Dragonfly High Speed Confocal Microscope System (Andor, Oxford Instruments, Abingdon, UK) located at the Microscopy Facility of the Institute of Functional Biology and Genomics (IBFG) (Salamanca, Spain) under the 40×/0.95 dry objective lens. A minimum of 15 FhNEJ were acquired per condition. Stacks of 31 slices (1 µm/slice) spanning the entire FhNEJ volume were acquired, and images were processed in FIJI software v.1.53t [55] by getting a maximum Z projection of each FhNEJ. For visualization purposes, histogram widths were equally adjusted in all conditions and the images were converted to RGB colour and exported as tiff files.

### 4.5. 2D Electrophoresis of FhNEJ-Teg

Additionally, 2D electrophoresis of FhNEJ-Teg was performed as previously described [14]. First, FhNEJ-Teg extract was purified using the ReadyPrep 2-D Cleanup Kit (BioRad, Hercules, CA, USA) and protein pellets were resuspended in rehydration buffer [7 M urea, 2 M thiourea, and 4% 3-[(3-cholamidopropyl) dimethylammonio]-1-propanesulfonate]. The protein extract was then divided in aliquots of 125 µL, supplemented with 0.05 M dithiothreitol (DTT) (Sigma, St. Louis, MO, USA), and 0.2% ampholytes pH 3–10 (BioRad, Hercules, CA, USA), and added to 7 cm ReadyStrip IPG Strips with a linear pH range of 3–10 (BioRad, Hercules, CA, USA) for passive rehydration overnight at 20 °C in a Protean Isoelectric Focusing (IEF) Cell equipment (BioRad, Hercules, CA, USA). IEF was performed the next day by using a constant amperage of 50 µA per strip in the Protean IEF Cell equipment (BioRad, Hercules, CA, USA). Next, the proteins in the strips were reduced with DTT (0.02 g/mL) and alkylated with iodoacetamide (0.0025 g/mL) for 10 min and 15 min, respectively, at room temperature (both DTT and iodoacetamide were diluted in equilibration buffer containing 6 M urea, 2% SDS, 1.5 M Tris-HCl pH 8.8, 30% glycerol, and bromophenol blue), and separation for the second dimension was completed in 12% acrylamide-sodium dodecyl sulphate (SDS) gels. These gels were stained with silver using in-house prepared reagents following the standard protocol (excluding formaldehyde and glutaraldehyde from the formulations to ensure compatibility with subsequent analysis by mass spectrometry) or transferred to a nitrocellulose membrane for immunoblot detection of LM binding. Silver-stained gels were imaged using a Chemidoc gel-imaging system (BioRad, Hercules, CA, USA).

### 4.6. Immunoblot Detection of LM-Binding Proteins

Immunoblot detection of LM-binding proteins was performed as previously described [14,51] with minor modifications. In brief, the proteins in the 2D gels were transferred to a nitrocellulose membrane using a constant amperage of 400 mA for 90 min at 4 °C. The blot was blocked for one hour at room temperature with 2% BSA diluted in PBST and incubated overnight at 4 °C with 5 µg/mL of LM (Santa Cruz Biotechnology, Dallas, TX, USA) diluted in blocking solution. LM binding was detected by adding an anti-LM primary antibody raised in rabbit (Abcam, Cambridge, UK) followed by HRP-conjugated anti-rabbit IgG (Sigma, St. Louis, MO, USA). Primary and secondary antibodies were diluted 1:2000 and 1:4000, respectively, in blocking solution and incubated for 90 min at 37 °C. Membranes were washed three times with PBST between antibody incubations and protein spots with bound LM were detected using enhanced chemiluminescence (Clarity Western ECL Substrate, BioRad, Hercules, CA, USA) on a ChemiDoc MP Imaging System (BioRad, Hercules, CA, USA). Spot matching between the silver-stained gel and the counterpart blot was performed using the PDQuest Software v.8.0.1 (BioRad, Hercules, CA, USA).

### 4.7. Spot Analysis by Liquid Chromatography Coupled to Tandem Mass Spectrometry (LC-MS/MS)

Proteomic analysis of LM-binding spots was performed as previously described [14]. Briefly, protein spots in the silver-stained gels were manually excised using 1000 µL sterile pipette tips and sent for proteomic analysis at the proteomics facility of the Central Support Service for Experimental Research (SCSIE, University of Valencia). In-gel protein digestion of every spot was performed using sequencing-grade trypsin (Promega, Madison, WI, USA) as described elsewhere [56]. In brief, 100 ng of trypsin were used for each sample, and digestion was performed overnight at 37 °C. Trypsin digestion was stopped with 10% trifluoroacetic acid (TFA) and the supernatant was removed. Next, samples were subjected to double acetonitrile (ACN) extraction and the peptide mixtures were dried in a speed vacuum and resuspended in 9 µL of 2% ACN, 0.1% TFA.

LC-MS/MS was carried out as follows: 5 µL of digested peptide mixtures were loaded onto a trap column (3µ C18-CL, 350 um × 0.5mm) (Eksigent, Dublin, CA, USA) and desalted with 0.1% TFA at 5 µL/min during five min. The peptides were then loaded onto an analytical column (3μ C18-CL 120 Å, 0.075 × 150 mm) (Eksigent, Dublin, CA, USA) equilibrated in 5% ACN plus 0.1% formic acid (FA). Elution was carried out with a linear gradient of 7–40% B in A for 20 min (A: 0.1% FA; B: ACN plus 0.1% FA) at a flow rate of 300 nL/min. Peptides were analysed in a mass spectrometer nanoESI qQTOF (6600plus TripleTOF) (ABSCIEX, Framingham, MA, USA). Samples were ionized in a Source Type: Optiflow < 1 µL Nano applying 3.0 kV to the spray emitter at 200 °C. Analysis was carried out in a data-dependent mode. Survey MS1 scans were acquired from 350–1400 *m*/*z* for 250 ms. The quadrupole resolution was set to ‘LOW’ for MS2 experiments, which were acquired 100–1500 *m*/*z* for 25 ms in ‘high sensitivity’ mode. The following switch criteria were used: charge 2+ to 4+, minimum intensity and 250 counts per second. Up to 100 ions were selected for fragmentation after each survey scan. Dynamic exclusion was set to 15 s. Finally, rolling collision energies equations were set for all ions as for 2+ ions according to the following equations: |CE| = (slope) × (*m*/*z*) + (intercept).

### 4.8. Protein Identification within LM-Binding Spots

The identification of proteins contained within LM-binding spots was performed as previously described [14]. Briefly, proteinPilot v5.0 search engine (ABSCIEX, Framingham, MA, USA) was used for protein identification. ProteinPilot default parameters were used to generate a peak list directly from 6600 plus TripleTOF wiff files and the Paragon algorithm [57] of ProteinPilot v5.0 was used to search the Uniprot_trematoda database (200604, 362615). The following parameters were used: trypsin specificity, IAM cys-alkylation, no taxonomy restriction, and search effort set to rapid analysis. Protein grouping was completed using the Pro Group algorithm (ABSCIEX, Framingham, MA, USA). Only proteins belonging to *F. hepatica* and having an Unused value equal to or greater than 2 were used for subsequent analyses, and protein isoforms were manually grouped to facilitate downstream data interpretation. Clades of cathepsin L entries with unspecified descriptions were assigned based on similarity to canonical cathepsin L prosegment sequences via multiple sequence alignment with Clustal Omega, and those corresponding to cathepsin B entries were determined by phylogenetic comparison of the entire protein sequence. GO analysis in the Biological Function category of LM-binding proteins identified via 2D-MS was performed using the software Blast2GO v5.2.

### 4.9. LM Degradation Assays

LM degradation by FhCL3 and by FhNEJ-Teg-induced plasmin generation from bound PLG were analysed. In the first setting, recombinant FhCL3 zymogen was activated in vitro by incubation for two hours at 37 °C in an appropriate volume of C-P buffer (0.1 M citrate-phosphate, 100 mM NaCl, 2 mM DTT, 10 µg/mL dextran sulfate, pH 4.5) [33] so that the final concentration of mature enzyme in the mix was 1 µM. In the second setting, 1.5 µg of FhNEJ-Teg were incubated in the presence or absence of 3 μg of PLG (Origene, Rockville, MD, USA) and 15 ng of u-PA (Sigma, St. Louis, MO, USA). In both instances, the reactions were performed in a total volume of 150 µL. A condition containing 1 µM of human plasmin (Origene, Rockville, MD, USA) was used at as a positive control for LM degradation [15]. The reactions mixtures were supplemented with 0.1 µg of LM (Santa Cruz Biotechnology, Dallas, TX, USA) and incubated at 37 °C for three hours. For visualization of LM degradation, equal amounts of samples were loaded after incubation into either in-house prepared 6% SDS-PAGE or pre-cast 4–20% Bis-Tris (GenScript, Piscataway, NJ, USA) gels and protein bands were revealed by silver stain or Sypro Ruby (Sigma, St. Louis, MO, USA) following standard procedures. Quantification of band intensity was completed by densitometry using FIJI software v.1.53t [55].

### 4.10. Analysis of LM-Induced Proteomic Changes in FhNEJ by SWATH-MS

In order to test whether LM interaction triggers proteomic changes in FhNEJ, we employed a methodology described by others [41,58,59] with minor modifications. First, 3 untreated 6 cm^2^ plates were coated with 76.44 μg LM (Santa Cruz Biotechnology, Dallas, TX, USA) diluted in 3 mL of coating medium (0.2 M NaHCO_3_, pH 9.5) overnight at 4 °C. Three uncoated plates were used as negative controls and incubated likewise in coating medium without LM. The next day, plates were washed 3 times with PBS and 400 FhNEJ per plate were added in Dulbecco’s Modified Eagle Medium (Capricorn Scientific, Ebsdorfergrund, Germany) supplemented with 2% foetal bovine serum (Capricorn Scientific, Ebsdorfergrund, Germany) and incubated for three hours at 37 °C in a 5% CO_2_ atmosphere. After incubation, FhNEJ were harvested and subjected to protein extraction for the isolation of FhNEJ-Teg and FhNEJ-Som as described in Section 4.2. 

Quantitation of protein concentration in all samples was performed using a detergent-compatible kit (Protein Quantification Assay; Machery-Nagel, Düren, Germany) following the manufacturer’s instructions. Prior to gel separation, 10 μg of each protein sample were diluted in an appropriate volume of 2× Laemmli Sample Buffer (BioRad, Hercules, CA, USA) supplemented with β-mercaptoethanol and were denatured at 95 °C for five min. Gel separation was performed using Any kD Mini-Protean TGX Precast Protein Gels (BioRad, Hercules, CA, USA) at 200 V for five min, followed by fixation in 40% ethanol/10% acetic acid for one hour and stained with colloidal Coomassie (BioRad, Hercules, CA, USA) for an additional one hour. After destaining in milliQ water, in-gel digestion of protein samples was performed with sequencing grade trypsin (Promega, Madison, WI, USA) as described elsewhere [56]. Specifically, gel bands were cut and digested using 500 ng of trypsin in 50 µL of 50 mM ammonium bicarbonate solution and digestion was stopped by addition of TFA to a final concentration of 1%. Peptides were then recovered by performing a double extraction with ACN, dried in a rotatory evaporator, and resuspended in the appropriate volume of 2% ACN/0.1% TFA to a final concentration of 0.3 μg/μL. 

For library construction, all *F. hepatica* samples were pooled together and loaded onto a trap column (3μ C18-CL 120 Ᾰ, 350 µm × 0.5 mm; Eksigent, Dublin, CA, USA) and desalted with 0.1% TFA at 5 µL/min for three min. The peptides were then loaded onto an analytical column (3μ C18-CL 120 Ᾰ, 0.075 × 150 mm; Eksigent, Dublin, CA, USA) equilibrated in 5% ACN/0.1% FA. Elution was performed over 60 min in a linear gradient of 7–40% solvent B in A (A: 0.1% FA; B: ACN, 0.1% FA) at a flow rate of 300 nL/min and analysed in a mass spectrometer nanoESI qQTOF (6600plus TripleTOF, ABSCIEX, Framingham, MA, USA). Eluted peptides were ionized applying 2.8 kV to the spray emitter. Analysis was carried out in a data-dependent mode. Survey MS1 scans were acquired from 350–1250 *m*/*z* for 250 ms. The quadrupole resolution was set to ‘UNIT’ for MS2 experiments, which were acquired 100–1500 *m*/*z* for 150 ms in ‘high sensitivity’ mode. Protein identification for library construction was performed with ProteinPilot v5.0 (SCIEX, Framingham, MA, USA) using default parameters to generate a peak list directly from 6600 tripleTOF wif files. The Paragon algorithm [57] was used to search a database containing the predicted proteome of *F. hepatica* (PRJEB25283, https://parasite.wormbase.org/Fasciola_hepatica_prjeb25283/Info/Index (accessed on 10 March 2022) appended to the cRAP contaminant database (https://www.thegpm.org/crap/ (accessed on 10 March 2022) [17]. 

For SWATH-MS analysis, 5 μL of each peptide mixture were loaded onto a trap column (NanoLC Column, 3μ C18-CL, 75 μm × 15 cm, Eksigent, Dublin, CA, USA) and desalted with 0.1% TFA at 2 μL/min during ten min. The peptides were then loaded onto an analytical column (LC Column, 3 μ C18-CL, 75 um × 12 cm, Nikkyo, Tokyo, Japan) equilibrated in 5% ACN/0.1% FA. Peptide elution was carried out with a linear gradient of 5 to 40% solvent B (A: 0.1% FA; B: ACN, 0.1% FA) for 90 min at a flow rate of 300 nl/min and analysed in a mass spectrometer nanoESI qQTOF (6600 TripleTOF, ABSCIEX, Framingham, MA, USA). The tripleTOF was operated in SWATH (DIA) mode, in which a 0.050-s TOF MS scan from 350–1250 *m*/*z* was performed, followed by 0.080-s product ion scans from 350–1250 *m*/*z* on the defined windows (3.05 s/cycle). We used 37 SWATH windows with 15 Da window width from 450 to 1000 Da. The wiff files obtained from SWATH individual acquisitions were analysed using PeakView 2.1 (SCIEX, Framingham, MA, USA). First, protein areas were calculated and normalized to the total sum of the areas of all the quantified proteins. Statistical treatment was performed using MarkerView 3.0 (SCIEX, Framingham, MA, USA), and evaluation of the differences between stimulated and control samples was performed using Student’s *t*-test coupled to Welch post hoc correction. Only proteins with a *p*-value < 0.05 were considered as differentially expressed. Discriminant Analysis with Pareto Scaling was performed for the reduction in dimensionality and plotted in R software, and protein descriptions and Uniprot accession numbers were retrieved using the software Blast2GO v6.0.3. Clades of cathepsin B entries were determined by phylogenetic comparison of the entire protein sequence.

### 4.11. Immunoblotting

Equal amounts of proteins from FhNEJ-Som samples used for mass spectrometry analysis were separated by SDS-PAGE in 12% gels and transferred to a nitrocellulose membrane. Total protein staining was performed with Sypro Ruby (Thermo Fisher Scientific, Waltham, MA, USA) according to manufacturer’s instructions followed by incubation for one hour at room temperature in a blocking solution of PBST containing 2% BSA. *F. hepatica* cathepsin B1-3 anti-serum produced in rabbit was diluted 1:500 in blocking buffer and added to the blot overnight at 4 °C. After incubation with HRP-conjugated secondary antibody (Sigma, St. Louis, MO, USA) diluted 1:2000 in blocking buffer, protein bands were detected using enhanced chemiluminescence (Clarity Western ECL Substrate, BioRad, Hercules, CA, USA) on a ChemiDoc MP Imaging System (BioRad, Hercules, CA, USA).

### 4.12. Statistical Analysis

Plots were created with Prism 9 software (GraphPad Software, La Jolla, CA, USA) and statistical analyses were performed with the R Commander package [60]. Comparison between three or more groups was completed with Analysis of Variance (ANOVA) test followed by Tukey post hoc correction for pair-wise comparisons. Asterisks indicate significant differences between the corresponding experimental groups (** *p* ≤ 0.01, *** *p* ≤ 0.0001). Unless otherwise stated, differences are not significant.

## 5. Conclusions

In conclusion, we have shown that the tegument-enriched antigenic fraction of FhNEJ contains an array of proteins that are capable of interacting with LM, a major component of the intestinal basement membrane. Among these, FhCL3 stands out provided the ability of this protease to degrade this important intestinal ECM component, an enzymatic function that is described for the first time in this study. Additionally, our results also demonstrate that the endogenous capacity of FhNEJ to degrade LM is further enhanced by co-opting the proteolytic activity of host plasmin. Finally, our results suggest that the interaction between FhNEJ and LM triggers intracellular signalling pathways that might be relevant during FhNEJ migration, development, and adaptation to the newly encountered host tissue microenvironment. Since the intestinal ECM functions as a physical barrier that blocks pathogen invasion, proteins that bind to and degrade ECM components shall be regarded as important virulence factors in *F. hepatica* that may be worth targeting for fasciolosis treatment and control during early infection.

## Figures and Tables

**Table 1:** DEPs identified by SWATH-MS in FhNEJ incubated in LM-coated plates as compared to their control counterparts and separated by antigenic compartment.

**FhNEJ-Teg DEPs**	**Uniprot Accession**	***p*** **-Value**	**Log2FC**
Alkylated DNA repair protein alkB 1	A0A4E0RF73	0.012	1.95
Calcium-binding mitochondrial carrier protein SCaMC-1	A0A4E0RTX3	0.037	1.76
Cargo transport protein erv29	A0A4E0RGI5	0.034	1.18
Golgi-associated plant pathogenesis protein 1	A0A2H1C4H4	0.044	1.01
Glutathione S-transferase omega class	A0A4E0RYR4	0.036	0.53
Propionyl-CoA carboxylase alpha chain	A0A4E0S3G7	0.012	−1.21
**FhNEJ-Som DEPs**	**Uniprot Accession**	***p*** **-Value**	**Log2FC**
Nuclear protein localization protein 4	A0A4E0R4V7	0.037	2.15
NADH dehydrogenase	A0A4E0R2Z1	0.012	2.10
Glucose-6-phosphate isomerase	A0A4E0RGH6	0.049	1.85
40S ribosomal protein S2	A0A4E0RD65	0.032	1.80
Programmed cell death protein 6	A0A4E0S2T2	0.023	1.71
Alpha-aminoadipic semialdehyde synthase	A0A4E0RQ3	0.008	1.70
60S ribosomal protein L6	A0A2H1BY45	0.010	1.66
Superoxide dismutase	A0A2H1CLW2	0.032	1.62
Eukaryotic ribosomal protein L18	A0A2H1C1P4	0.027	1.46
Thioredoxin peroxidase	A0A4E0RVH8	0.038	1.44
Cathepsin B2	A0A4E0RNG2	0.027	1.41
Solute carrier family 2 facilitated glucose transporter member 3	A0A4E0RGV2	0.042	1.37
Spectrin beta chain	A0A4E0RR49	0.035	1.19
Heat shock protein 70	B1NI98	0.034	0.99
Oxoglutarate dehydrogenase	A0A4E0RQS7	0.044	−1.22

## Data Availability

Not Applicable.

## Supplementary Materials

The following supporting information can be downloaded at: https://www.mdpi.com/article/10.3390/ijms24098165/s1.
