# Supplementary figures and images for: Molecular Characterization of the Interplay between Fasciola hepatica Juveniles and Laminin as a Mechanism to Adhere to and Break through the Host Intestinal Wall
Source: Int J Mol Sci. 2023 May 3;24(9):8165. doi: 10.3390/ijms24098165 (PMC10179147; doi:10.3390/ijms24098165)

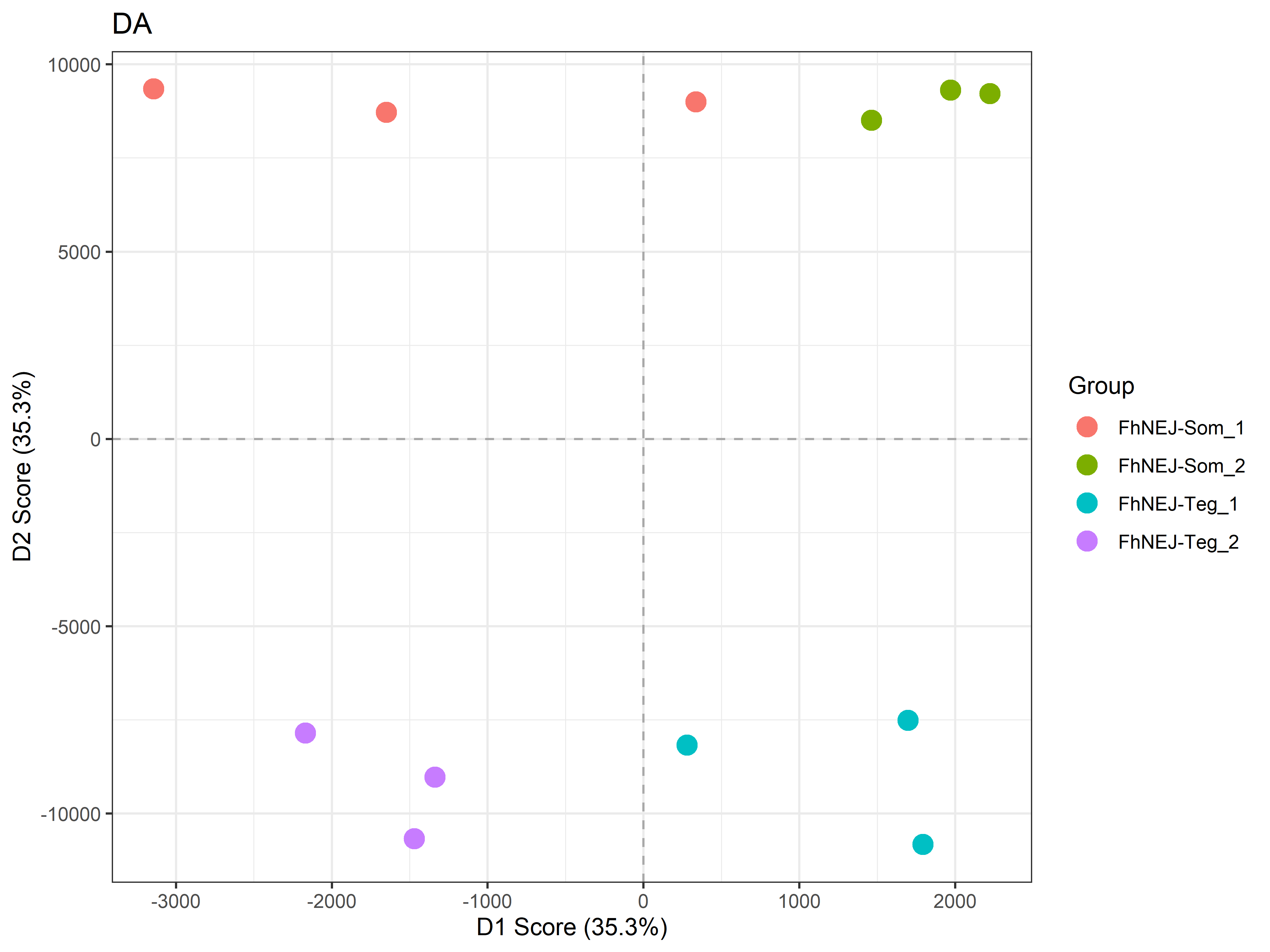

Supplement: Supplementary file 1 [file ijms-24-08165-s001.zip › Figure S2.tif]
